# Recognition of Differentially Expressed Molecular Signatures and Pathways Associated with COVID-19 Poor Prognosis in Glioblastoma Patients

**DOI:** 10.3390/ijms24043562

**Published:** 2023-02-10

**Authors:** Faisal A. Alzahrani, Mohd Faheem Khan, Varish Ahmad

**Affiliations:** 1 Department of Biochemistry, Faculty of Science, Embryonic Stem Cell Unit, King Fahad Center for Medical Research, King Abdulaziz University, Jeddah 21589, Saudi Arabia; 2 Department of Biotechnology, Khandelwal College of Management Science and Technology (KCMT), Mahatma Jyotiba Phule Rohilkhand University, Bareilly 243006, India; 3 Health Information Technology Department, The Applied College, King Abdulaziz University, Jeddah 21589, Saudi Arabia; 4 Centre of Artificial Intelligence for Precision Medicines, King Abdulaziz University, Jeddah 21589, Saudi Arabia

**Keywords:** hub genes, COVID-19, GBM, co-expression network, protein–protein interaction network

## Abstract

Glioblastoma (GBM) is a type of brain cancer that is typically very aggressive and difficult to treat. Glioblastoma cases have been reported to have increased during COVID-19. The mechanisms underlying this comorbidity, including genomic interactions, tumor differentiation, immune responses, and host defense, are not completely explained. Therefore, we intended to investigate the differentially expressed shared genes and therapeutic agents which are significant for these conditions by using in silico approaches. Gene expression datasets of GSE68848, GSE169158, and GSE4290 studies were collected and analyzed to identify the DEGs between the diseased and the control samples. Then, the ontology of the genes and the metabolic pathway enrichment analysis were carried out for the classified samples based on expression values. Protein–protein interactions (PPI) map were performed by STRING and fine-tuned by Cytoscape to screen the enriched gene module. In addition, the connectivity map was used for the prediction of potential drugs. As a result, 154 overexpressed and 234 under-expressed genes were identified as common DEGs. These genes were found to be significantly enriched in the pathways involved in viral diseases, NOD-like receptor signaling pathway, the cGMP-PKG signaling pathway, growth hormone synthesis, secretion, and action, the immune system, interferon signaling, and the neuronal system. STAT1, CXCL10, and SAMDL were screened out as the top 03 out of the top 10 most critical genes among the DEGs from the PPI network. AZD-8055, methotrexate, and ruxolitinib were predicted to be the possible agents for the treatment. The current study identified significant key genes, common metabolic signaling networks, and therapeutic agents to improve our perception of the common mechanisms of GBM–COVID-19.

## 1. Introduction

Glioblastoma (GBM) is one of the most dangerous diseases and is one in which the brain and spine are affected. Due to its rapid spread, its survival rate is very low [1]. Therefore, there is a need for continuous research in this direction. Glioblastoma can have a substantial impact on patients’ brain and immune systems. As a result, any additional disease in these people can reduce their chances of survival [1,2]. Therefore, there is a need to protect the glioblastoma patient from other diseases. A recent study suggested that the boost in GBM due to viral infection and its high mortality rate might because of a lack of specific treatment [2]. The survival status of patients during the viral–glioblastoma association has already been proven by using in vitro xenograft models [3]. It has already been found that the IDH1 mutation is known to be a major cause of GBM. Recently, a different genetic signature was found, but the reports that were out there did not say how this signature was divided [4], whereas COVID-19 is a viral disease that originated in China in late 2019 and, by October 2020, had spread to the entire world. People of all age groups were affected almost all over the world. In most cases, immunocompromised people in their forties and fifties were found to have a more severe disease [5]. The current research has also suggested that people with cancer may be at a higher risk for the disease. Patients with GBM spend a lot of time exposed to chemotherapy, radiotherapy, and other drugs [6]. The side effects of these treatments have been reported in immunocompromised patients. These patients are more vulnerable to coronavirus disease and its complications. The health of patients with GBM is extremely vulnerable to the COVID-19 infection. According to a particular cohort study, these diseases cause a higher mortality rate in GBM patients than in those with benign illnesses. With the long-term consequences of the previous pandemic in mind, new policies and management are urgently needed to avert it. The detection of disease-specific drugs for the management of virally infected GBM patients requires advanced genomics and metabolomics data mining approaches [7]. The present research aims to explore the interconnection between GBM and COVID-19. The latest research advances, such as metabolic interaction, drug interaction, bioinformatics, high-throughput sequencing, the presence of data repositories, such as gene expression microarray data, and next-generation sequencing, have made it possible to detect various diseases and the linkages between them. The hypoxic condition has a strong association with COVID-19, and the effect that has been observed in GBM is highly deleterious. It is not yet fully understood why the mortality rate of GBM patients became higher during the pandemicand what the connection is between the GBM and the COVID-19 mechanisms and the associated consequences. There is still a lack of sufficient information to understand this lethal combination. In the present study, we took the GSE68848, GSE169158, and GSE4290 studies, two of which are differential gene expression studies of GBM (GSE68848, GSE4290) and one of whichis a study of the coronavirus2 disease. The information obtained from the presented research will be of great benefit to the entire research community for the better treatment of this comorbidity.

## 2. Results

### 2.1. Identification of Common Differentially Expressed Genes between GEO Studies

**Figure 1:**
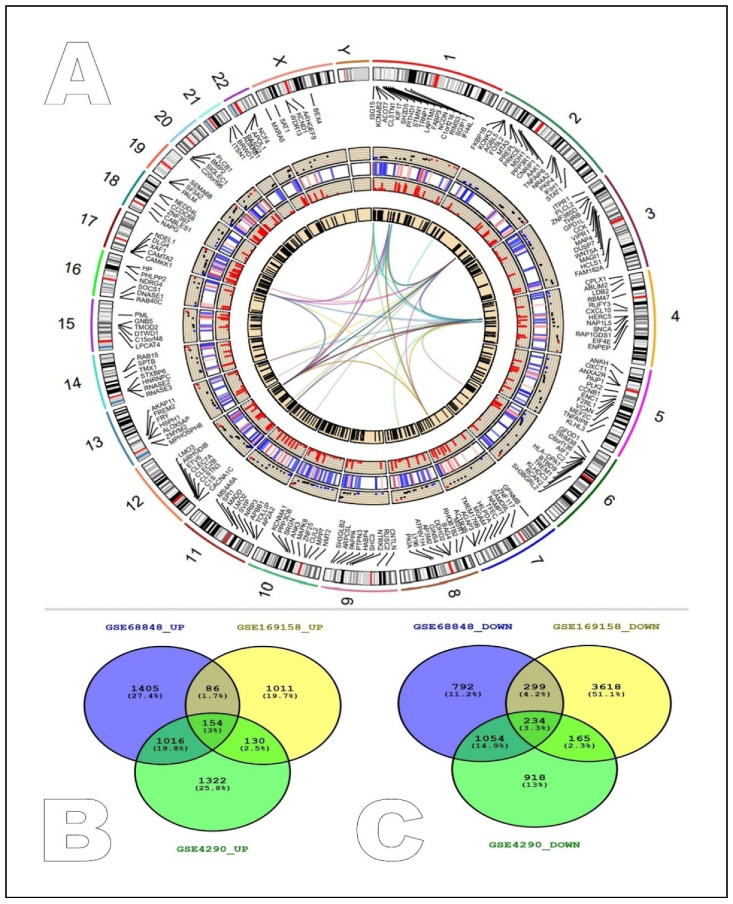
Distribution and status of shared differentially expressed genes and the intersection of yellow, black, and blue modules. (**A**) Circos layout: First circle: characterization of the human genome based on chromosome numbers. Second circle: locations of up-regulated genes (red), down-regulated (blue), and control genes (black) in all 24 chromosomes. Third circle: status of differentially expressed genes. Fourth circle: co-expression of up- and down-regulated genes. Fifth circle: only up-regulated genes. Core region: multiple color lines depicting the interaction between hub genes. (**B**) Shared up-regulated genes between studies. (**C**) Shared down-regulated genes between selected studies.

The distribution, characterization, and interaction of the overexpressed genes (red), down-regulated genes (blue), and control genes (black) were represented through the Circos layout. The Circos plot’s first track depicts the approximate length of each of the 24 human chromosomes, including the X and Y chromosomes. The second track further categorizes the length of the chromosomes based on the expression of certain genes across the entire genome. The fourth track shows the abnormally overexpressed genes, while the third track represents the down- and intermediately expressed genes. As a representational entity for the control genes that were found to remain intact during the diverse disease scenarios, the fifth circle or track was represented by a black bar (Figure 1A). An analysis of the COVID-19 and GBM patients against the control samples (normal individuals) in the GSE16958, GSE68848, and GSE4290 datasets was performed to identify the common signatures of the differentially expressed genes using *p*-value < 0.01 and the Log2 fold change 1 criterion. The comparison revealed that the GSE68848 (glioblastoma) contained 6516 DEGs, 3325 of which were overexpressed and 2379 of which were low-regulated (Supplementary_File1), whereas in the dataset GSE4290, 1381 genes were found to be overexpressed, and 4316 genes were found to be down-regulated. The GSE69158 (COVID-19) RNAseq study was analyzed with Galaxy servers and 3710 overexpressed and 2371 low-regulated genes were found. The shared signatures were found as 154 up-regulated genes and 234 down-regulated genes between the selected studies. This shared up and down pattern can be identified as the perfect signature for the common metabolic pathways between these two diseases (Figure 1B).

### 2.2. Functional Analysis of Differentially Expressed Genes

DAVID and KEGG pathway analyses were used to identify the important gene ontology for the evaluation of the functions of the identified differentially expressed genes. The analyses revealed that the DEGs were found to be enriched in the important biological processes between glioblastoma and COVID-19. The positive regulation of the immune system processes and immune responses, the defense responses to other organisms, the negative regulation of trans-synaptic signaling, and the chemical synaptic transmission (Table 1) were found to be key biological processes. Enriched cytoplasmic vesicles, intracellular vesicles, secretary granules, synapses, and cell junctions were identified as major cellular components (Table 1). 2′-5′-oligoadenylate synthetase activity, Toll-like receptor 4 binding, arachidonic acid binding, cytoskeleton protein binding, and the nucleoside-triphosphatase regulator were the enriched cellular components involved in the COVID-19–GBM interconnection network activity (Table 1).

Based on the KEGG pathway analysis, it was found that the DEGs were enriched in influenza A, coronavirus disease—COVID-19, the NOD-like receptor signaling pathway, the cGMP-PKG signaling pathway, and the growth hormone synthesis, secretion, and action pathway. The Reactome pathway analysis revealed that the DEGs were enriched in the immune system, the interferon signaling, the innate immune system, and the neuronal system signaling by receptor tyrosine kinase (Table 2).

**Figure 2:**
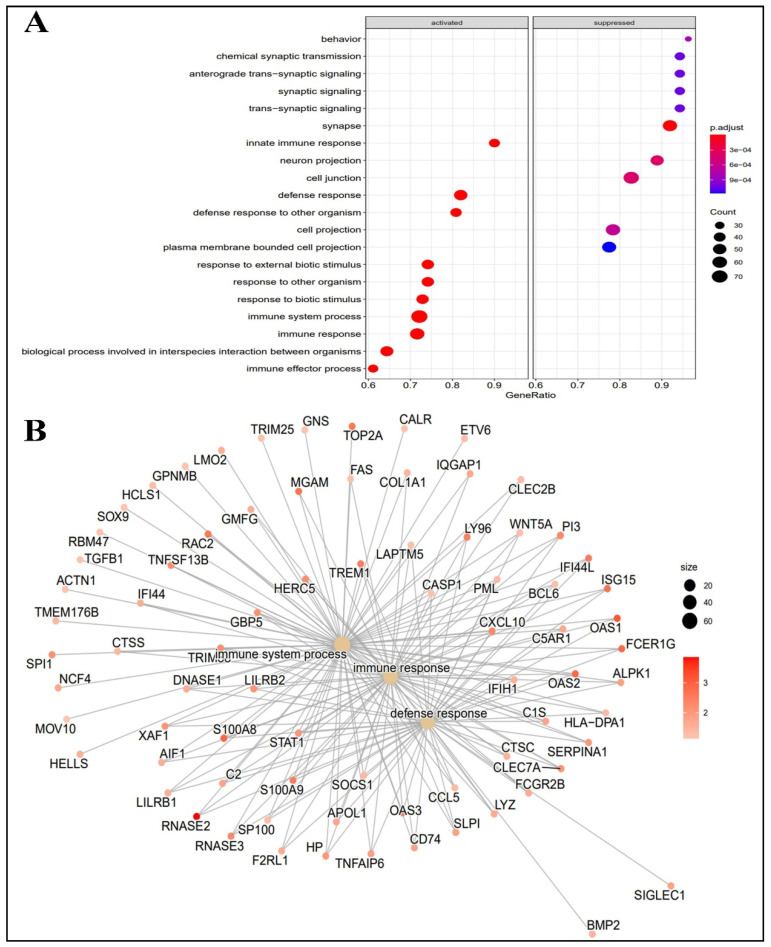
(**A**) Profiling of biological processes, molecular function, cellular components, and their expression status (activated, suppressed), (**B**) Major biological processes and response representation according to their fold change value, greater diameter, and darkness of color indicated major involvement between GBM and COVID-19 comorbidity.

The profiling of the biological processes indicated that the innate immune response, the defense response, the defense response to another organism, the response to an external biotic stimulus, the response to other organisms, the response to a biotic stimulus, the immune system processes, the immune responses, and the biological process between interspecies interaction and the immune effector responses were activated, and behavior, chemical synaptic transmission, anterograde-transsynaptic signaling, synaptic signaling, synapse and neuron projection, cell junction, cell projection, and plasma membrane-bound cell projection were found suppressed during infection withCOVID-19 in GBM patients (Figure 2A). The GSEA also revealed that most of the DEGs have major involvement in the immune system processes and the immune process and defense responses (Figure 2B).

### 2.3. PPI Network Construction and Modules Selection

**Figure 3:**
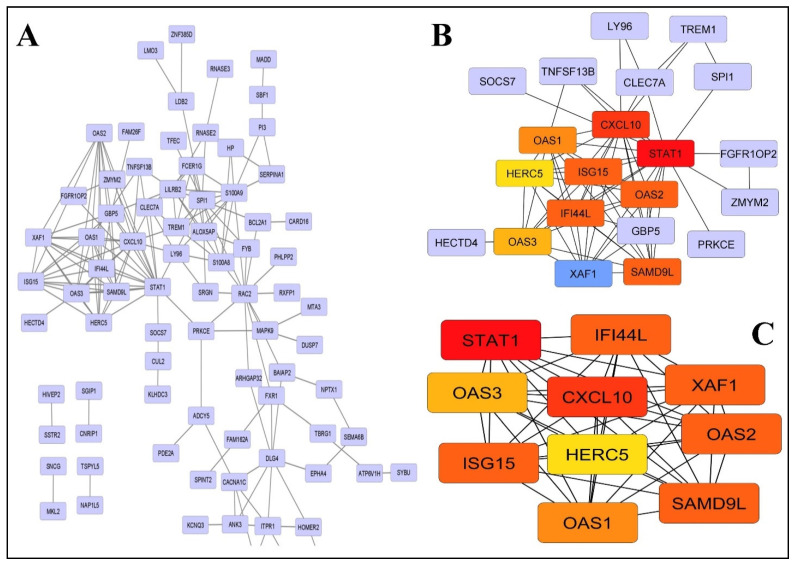
Protein–protein interaction network of DEGs. (**A**) A total of 203 nodes and 346 interaction associations were identified. The nodes with the highest PPI scores were shaped as the diamond in yellow. (**B**) The most significant module from the PPI network and their best nearby interaction. (**C**) Important Hub genes.

A protein interaction network was developed for differentially expressed genes through the STRING database (version 11.0). The constructed network has164 nodes and 228 edges (Figure 3A). The significant hub genes with the highest protein interaction score were identified by four centrality methods, with degree 10 as the cut-off criterion (Table 3). However, the Matthews correlation coefficient method was considered for further analysis as it was found to have better performance in the prediction accuracy of essential genes (Table 3).

Genes such as Signal Transducer and Transcription-1 (STAT1), CXC motif chemokine ligand-10 (CXCL10), Sterile Alpha Motif Domain Containing-9 like (SAMD9L), X-Linked Inhibitor of Apoptosis (XIAP), XIAP Associated Factor-1 (XAF1) Interferon Induced Protein-44 like (IFI44L), 2′-5′-Oligoadenylate Synthetase-2 (OAS2), Interferon Stimulated Gene-15 (ISG15), 2′-5′-oligoadenylate Synthetase 1 (OAS1), and Oligoadenylatesynthetase-3 (OAS3) were identified as key hub genes. These genes are further represented by light to dark shades of orange color according to their low to high expression intensity. Among these genes STAT1 was identified as the most influential gene 28 with the highest node degree (Figure 3B).

The protein module was developed by the PPI network of DEGs with the help of MCODE, consisting of 10 nodes and 45 edges (Figure 3C). The biological functional enrichment analysis revealed that the identified genes were significantly enriched in the 2′-5′-oligoadenylate synthase activity, adenyl-transferase activity, double-stranded RNA binding, and nucleotidyltransferase activity (Table 4). These functions were found to be significantly correlated with COVID-19, the NOD-like receptor signaling pathway, and the antiviral mechanism because of the involvement of IFN-stimulated genes. Interferon signaling and cytokine signaling were the two major types of signaling that showed the greatest enrichment evidence through KEGG pathway analysis.

### 2.4. cMAP Analysis

All of the differentially expressed and hub genes were analyzed by the CMAP server. The top 10 compounds with low connectivity scores (0.57 to 0.50 for DEGs) and (−0.89 to −0.78 for selected hub genes) were identified (Table 5). These compounds (i.e., bexarotene, cisapride, idebenone, hydroflumethiazide, sulbutiamine, methimazole, butorphanol-(+)-tartrate, nefiracetam, melatonin, and lapatinib in the case of the total and the differentially expressed genes and linezolid, erastin, enalapril, AZD-8055, carbamazepine, BX-795, methotrexate, eugenol, tolterodine, and selumetinib) may act as significant therapeutic candidates to reverse or counteract the abnormal gene expression and could be used as promising novel therapies.

### 2.5. Statistical Analysis

Differential gene expression analyses were performed based on log2FC| > 2 and *p* < 0.05 as statistically significant parameters. In addition, most of the statistical values were obtained by the default statistical methods used by the different bioinformatics tools involved in the workflow of the current research work.

## 3. Discussion

People with glioblastoma have been observed to experience significant changes in their immune systems. The abrupt drop in the T-cell population is the most notable of these alterations [8]. The connected connection between our brain and immune system is to blame; our auxiliary pro-inflammatory cytokines help to govern this process [9]. Immunogenetic markers, which are in charge of controlling innate immune responses, are also closely connected to mortality in patients with GBM, COVID-19, and poor prognosis [10,11]. A high death rate in GBM patients with a severe COVID-19 infection is mostly caused by the suppression of the innate immune system [12]. It has also been found that innate immunity is very important in severe COVID-19 infections, which means that targeted therapies are needed [13]. Notably, the T-cell response is an important component of immunological memory and may be a characteristic vaccine formulation technique. The molecular signature discovered between the two diseases in the current investigation further supports the above discussion.

The analysis of the selected gene expression datasets (GSE68848, GSE169158, and GSE4290) also suggested the importance of the innate immune response between GBM and COVID-19. Despite advances in modern molecular biological research and therapeutics, the metabolic interaction mechanisms underlying COVID-19andGBM have not been fully understood. In the present study, the KEGG and Reactome pathways compared the differentially expressed genes of COVID-19 and GBM. Through this analysis, it was found that these genes were involved in many different biological processes, such as viral infections, inflammation caused by tumors, and immune responses. It has been proven that viral infection can initiate onco-modulation in brain tumors [14,15]. It was revealed that the genes related to COVID-19 were found to be overexpressed in the NOD-like receptor signaling pathway, interferon signaling, and the innate immune system, whereas the genes related to the cGMP-PKG signaling pathway were found to be down-regulated. The up-regulation of the NOD-like receptors is associated with poor prognosis, higher epithelial-mesenchymal transition (EMT) signaling, and proliferation in GBM patients [14,16,17]. EMT is a reversible biological process occurring in the epithelium. EMT eventually leads to the acquisition of a mesenchymal phenotype, which reflects increased cell motility and resistance to genotoxic agents [18]. These processes are mostly accompanied by the acquisition of stem cell properties in differentiated tumor cells and are necessary in enabling carcinoma cells to repress their epithelial characteristics by turning mesenchymal [19,20]. This allows the cells to have motility and the ability to migrate from the primary site. The N protein of SARS-CoV-2 also induces hyper-inflammation through the NOD-like receptor, which may be an important factor in GBM acceleration [21]. The higher expression of interferon signaling was shown as an unfavorable prognostic factor in GBM [22,23,24]. The ssRNA genome and the dsRNA replication intermediates of SARS-CoV-2 can be sensed by Toll-like receptors (TLRs) and retinoic acid-inducible gene-I-like receptors (RLRs) in host cells. These TLRs and RLRs can activate the interferon response via the transcription factors NfKB and IRF3/7 [25]. Though the IFN response decreases in moderate COVID-19 patients, a paradoxically higher IFN response is associated with severe COVID-19 disease [26,27]. This implies that GBM patients can be at an elevated risk of severe COVID-19. The SARS-CoV-2 infection can activate innate immune cells to release inflammatory cytokines such as interleukin-1, interleukin-6, interleukin-8, interleukin-12, tumor necrosis factor-alpha, interferon gamma, CCL2, granulocyte colony-stimulating factor, granulocyte-macrophage colony-stimulating factor, and other cytokines or chemokines [28]. The induction of these cytokines is associated with cytokine syndrome in COVID-19 patients. GBM patients may have a chronic innate immune response activated, making them more vulnerable to cytokine syndrome. The cGMP/PKG pathway is found to be significantly down-regulated in GBM. The pharmacological activation of this pathway can inhibit glioma cell proliferation. The down-regulation of the cGMP/PKG pathway may induce aggressive glioma [29]. SARS-CoV-2 can induce ROS, which can lead to a reduction in nitric oxide, resulting in a low cGMP/PKG pathway [30]. Thus, the SARS-CoV-2 infection may worsen glioma progression.

The GO analysis results indicated that the DEGs were enriched in biological processes related to the activation of immune responses and down-regulatingsynaptic signaling. It is known that glioma cells can induce neurodegeneration by down-regulating synapses [31], whereas COVID-19 directly affects multiple regions of the brain, including direct infection of the neural cells and severe systemic inflammation, which deluge the brain with pro-inflammatory agents and thereby injure nervous cells. As a result, the SARS-CoV-2 infection is also suspected of causing neurodegeneration and irreversible neuronal damage [31].

A protein interaction network was developed for the differentially expressed genes and identified the top 10 hub genes. The gene ontology analysis suggested that the identified genes were related to the antiviral defense response. During a viral infection, the genitourinary defense system is the first line of defense. These adaptive responses are then necessary to direct the vital components responsible for the development and protection of health. STAT1 activation is dependent on Syk rather than cytokine-activated JAK signaling at the early stage of viral infection, which is important for early antiviral immunity [32].

The chemokine-to-cytokine-to-chemokine cascade (CXCL2) is an important lethality which is essential for defense during viral infections that establish themselves in tissues [33]. Whereas SAMD9L is a major component of the hallmark antiviral type-1 interferon response of many cells, it shows a multifold increase following stimulation with messenger RNA (mRNA) interferon beta. The down-regulation of p53 has been observed in the absence of XAF1, suggesting that XAF1 modulates p53 activation during VSV infection. Therefore, further research on XAF1 may lead to a new link between cancer and viral infection. The links may open new avenues for its treatment [34]. IFI44L is known to be a better option for the treatment of diseases associated with excessive IFN levels and proinflammatory responses and to reduce viral replication [35]. Various members of the OAS family are known to be critical for controlling DEN replication in human cells; an innate immune pathway of the 2′,5′-oligoadenylate synthatase (OAS)/RNase L system inhibits viral infection by responding to a pathogen-associated molecular pattern to induce the degradation of cellular RNA [36]. Therefore, these may be important agents for COVID-19-infected GBM individuals. ISG15 is known to directly prevent viral replication, and it has been shown that ISG15 plays a very important role in controlling the host damage, repair response, immune response, and other host signaling pathways [37]. The E3 ligase activities of HERC5 have been identified in a variety of biological processes, such as protein degradation, cell signaling, tumor suppression, and antiviral defense. Therefore, it is a very good gene family for research exploration into COVID-19–GBM comorbidity [38].

Drug mining was performed for the total differentially expressed and important hub genes. The top 10 potential compounds with negative connectivity scores, including linezolid, erastin, enalapril, AZD-8055, carbamazepine, BX-795, methotrexate, eugenol, tolterodine, and selumetinib, were selected. These compounds showed efficiency in reversing the DEG trends. Out of the total selected compounds, AZD-8055 and methotrexate are of particular interest in our study. The PI3K/Akt/mTOR pathway is found to be activated in almost 90% of all GBM cases [39]. AZD-8055 is a MTOR kinase inhibitor that can inhibit both AKT and MTOR signaling [40]. DNA or RNA viruses can activate the PI3K/AKT/MTOR pathway, and MTOR inhibition can suppress viral protein synthesis [41]. The SARS-CoV-2 infection is shown to increase MTORC1 by rewiring the host cell metabolism. Thus, inhibiting MTOR signaling can target both the GBM and the SARS-CoV-2 viral loads.

Methotrexate has been used as first-line therapy for rheumatoid arthritis (RA) for the last 40 years. RA is an autoimmune and inflammatory disease. Methotrexate is anti-inflammatory and can decrease cytokine profiles. Methotrexate can increase adenosine levels, which in turn activates the adenosine receptors. The activated adenosine receptors can promote an overall anti-inflammatory state. Thus, methotrexate use can reduce the severe cytokine syndrome profile of COVID-19andcan be selectively toxic for glioma stem cells by targeting folate metabolism [42].

From a drug design standpoint, hub genes and their associative networks are always of interest; thus, the mining of essential agents that can reverse the abnormal trend during COVID-19–GBM comorbidity was also performed, and 10 essential agents were screened for counteracting the disease condition. The compounds that were looked at might be able to change how hub genes talk to each other and how their network works as a whole. Roxolitinib, lenalidomide, pazopanib, PF-04457845, PRT-062070, Trapidil, Eugenol, SR-59230a, and UNC-669 were the top drugs discovered using this method. Out of these drugs, ruxolitinib was of particular interest because of its high negative correlation value. It is a JAK2-specific inhibitor and thus can down-regulate JAK-STAT signaling. Many patients, including those with COVID-19 and the heavy inflammatory syndrome, were successfully treated with ruxolitinib [42], and it was also safe for GBM patients. In a case study, COVID-19-associated ARDS was also successfully treated using ruxolitinib [43]. Patients with GBM who received ruxolitinib in addition to temozolomide and radiation had significantly better OS and PFS than those who only received temozolomide and radiation [44]. Linezolid is also a good way to treat COVID-19 patients who have bacterial nosocomial pneumonia. Pieces of evidence suggested that COVID-19 patients who were suffering from bacterial pneumonia and receiving an intravenous dose of 600 mg of linezolid every 12 h for 7 to 10 days recovered and were discharged from the hospital.

It has been reported that AZD-8055 is a powerful, selective, and orally accessible ATP-competitive mammalian target of rapamycin kinase inhibitor with anticancer activity, and so, it may be a promising treatment for GBM patients [40]. However, methotrexate belongs to a group of drugs called antimetabolites. Methotrexate cures cancer by reducing the growth of cancer cells. The same medication was also utilized during COVID-19 and showed noteworthy effects in cytokine storm scenarios. In light of the current study, we can therefore consider methotrexate a reasonable option [45]. Ruxolitinib, the third most-screened medicine, is known for inhibiting JAK1 and JAK2 and thereby inhibiting tumor invasion and tumorigenesis in human GBM, and it was also employed during COVID-19 for combined antiviral and anti-inflammatory therapy [46,47]. As a result, these medications already demonstrated their efficacy during the pandemic in patients who were already suffering from various ailments. In order to effectively personalize treatment, additional study is therefore required to explore the efficacy of these drugs.

## 4. Materials and Methods

### 4.1. Data Collection and Processing

The GSE68848, GSE169158, and GSE4290 gene expression datasets were collected from the NCBI-GEO [48]. These expression datasets were selected because of the clear classification between the diseased and the control genes for the respective diseases. GSE68848 has a total of 580 samples, which were categorized as mixed, GBM (diseased), oligodendroglioma, astrocytoma, unknown (uncategorized), and non-tumor (control). Only the diseased and control samples were considered in our study. The SARS-CoV-2 viral infection versus the mock infection dataset was obtained from GSE169158, and the DEGs were identified with the Galaxy server [49]. The GSE4290 study collected 23 samples from normal patients as controls and 81 samples from patients with diseased glioblastomas. The analysis of the common DEGs between the diseased and the control samples was conducted with the GEO2R package (LIMMA, Linear Models for Microarray Data). Moreover, the screening of the DEGs was performed with a 0.05 *p*-value as a threshold.

### 4.2. Enrichment Analysis of Genes and Pathways of Differentially Expressed Genes

Gene ontology enrichment and KEGG pathway [50] analysis were carried out by DAVID (Database for Annotation, Visualization, and Integrated Discovery) and GO-profiler (gene ontology analysis module developed in R) [51].

### 4.3. Construction of PPI Network and Selection of Hub Genes

The STRING app and Cytoscape were used in association to construct the PPI network and its visualization. MCODE (Molecular Complex Detection, a plugin for Cytoscape) was applied to screen the significant modules of the protein–protein interaction [52]. The parameters were set as confidence score > 0.4, cut-off degree = 2, cut-off node score = 2, k-core = 2, and maximum depth = 100 to evaluate the significant interaction between the DEGs. Hub genes have a high correlation in candidate modules that might be involved in important biological processes, and they were screened and investigated through MCC (maximal clique centrality), looseness, degree, and bottleneck approaches [53]. The MCC method was considered the most significant method to identify hub objects in this study because it could capture more essential genes in comparison to other methods in the top-ranked list of both high- and low-degree genes. As a result, the top ten hub genes identified by the MCC method were investigated further in the current study.

### 4.4. Connectivity Map (cMAP)

The cMAP database was used to find potential agents by comparing the identified molecular signatures of the differentially expressed genes associated with COVID-19–GBM comorbidity. Small therapeutic molecules with negative connectivity enrichment scores were selected based on >0.05 as a cut-off criterion [44].

## 5. Conclusions

The presented study provides some significant insights into the mechanisms underlying the impact and risk of SARS-CoV-2 infection on GBM patients. The COVID-19mortality rate was found to be much higher in patients with comorbidities as compared to the SARS-CoV-2 infection. This fact suggests that, in this comorbidity (GBM–COVID-19), it would be preferable for each to reuse disease-specific drugs that do not adversely affect the other’s metabolic pathways. The differentially expressed genes were screened and selected at the intersection of theCOVID-19and GBM expression studies. Their potential functions were annotated by GO analysis and pathway analysis. The DEGs were mostly enriched in the viral infection pathways and the tumor-induced inflammation and immune response pathways, and it was found that these pathways have significant interactions with the mechanisms of GBM. Subsequently, several key hub genes that may play an important role in COVID-19–GBM were demonstrated by PPI analysis.

The one-drug, one-target, and one-disease approach is less common these days. However, given the rapidity with which the negative effects of multi-target medications were observed in people already suffering from pandemic diseases, only single-target, disease-specific drugs can be effective in these circumstances. A total of 19 drugs have been identified that can reverse the tendency towards abnormal expression during disease states without affecting each other. AZD-8055, methotrexate, and ruxolitinib may have more promising potential in this regard. This study may provide a valuable clue for the treatment research on GBM patients experiencing COVID-19infection and on its prevention.

## Figures and Tables

**Table 1:** Gene ontology signatures of differentially expressed genes associated with SARS-CoV-2–GBM association.

GO Category	Term Name	Genes Enriched	%	*p*-Value
MF	Positive regulation of 2′-5′-oligoadenylate synthetase activity	3	2.1	1.25 × 10^−3^
	Positive regulation of Toll-like receptor 4 binding	3	2.1	1.25 × 10^−3^
	Positive regulation of arachidonic acid binding	3	2.1	6.17 × 10^−3^
	Negative regulation cytoskeleton protein binding	32	14.55	1.00 × 10^−4^
	Negative regulation of nucleoside-triphosphatase regulator activity	22	10	1.10 × 10^−4^
BP	Immune system process	76	57.58	1.26 × 10^−19^
	Immune response	62	46.97	1.55 × 10^−16^
	Defense response to other organisms	44	33.33	1.88 × 10^−16^
	Regulation of trans-synaptic signaling	32	15.09	1.78 × 10^−13^
	Chemical synaptic transmission	38	17.92	7.34 × 10^−12^
CC	Cytoplasmic vesicle	45	31.25	1.66 × 10^−6^
	Intracellular vesicle	45	31.25	1.77 × 10^−6^
	Secretory granule	24	16.67	8.60 × 10^−6^
	Synapse	64	29.22	1.08 × 10^−20^
	Cell junction	71	32.42	6.38 × 10^−15^

**Table 2:** KEGG and Reactome pathway analysis of the differentially expressed genes associated with SARS_CoV-2–GBM association.

Pathway Source	Pathway Name	Genes Enriched	%	*p*-Value
KEGG	Influenza A	13	14.94	2.35 × 10^−6^
KEGG	Coronavirus disease—COVID-19	11	12.64	3.15 × 10^−3^
KEGG	NOD-like receptor signaling pathway	9	10.34	1.02 × 10^−2^
KEGG	cGMP-PKG signaling pathway	12	12.9	3.57 × 10^−5^
KEGG	Growth hormone synthesis, secretion, and action	10	10.75	8.91 × 10^−5^
Reactome	Immune system	56	54.9	3.22 × 10^−13^
Reactome	Interferon signaling	15	14.71	1.60 × 10^−7^
Reactome	Innate immune system	33	32.35	3.53 × 10^−7^
Reactome	Neuronal system	18	14.06	4.87 × 10^−4^
Reactome	Signaling by receptor tyrosine kinases	19	14.84	1.93 × 10^−3^

**Table 3:** Top 10 differentially expressed genes with their weight scores based on four centrality methods.

MCC			Closeness			Degree			Bottleneck		
Rank	Name	Score	Rank	Name	Score	Rank	Name	Score	Rank	Name	Score
1	STAT1	367,933	1	STAT1	35.93333	1	STAT1	17	1	RAC2	52
2	CXCL10	367,932	2	SPI1	35.91667	2	CXCL10	14	2	SPI1	43
3	SAMD9L	367,920	3	RAC2	34.78333	3	SPI1	13	3	STAT1	33
3	XAF1	367,920	4	S100A9	32.45	3	RAC2	13	4	PRKCE	14
3	IFI44L	367,920	5	CXCL10	31.13571	5	S100A9	11	5	BAIAP2	13
3	OAS2	367,920	6	LILRB2	30.66667	6	OAS3	10	6	DLG4	11
3	ISG15	367,920	7	S100A8	30.2	6	SAMD9L	10	7	ADCY5	10
8	OAS1	362,886	8	PRKCE	29.93333	6	XAF1	10	7	S100A9	10
9	OAS3	362,881	9	TREM1	29.08333	6	OAS1	10	9	CACNA1C	8
10	HERC5	362,880	10	GBP5	28.71905	6	IFI44L	10	10	FXR1	6

**Table 4:** Gene ontology enrichment classification of selected hub genes module.

Category	Term/Function	Genes Enriched	Percentage	*p*-Value
Molecular function	2′-5′-oligoadenylate synthetase activity	3	30	7.83 × 10^−8^
	Adenylyltransferase activity	3	30	8.74 × 10^−5^
	Double-stranded RNA binding	3	30	1.46 × 10^−3^
	Nucleotidyltransferase activity	3	30	7.40 × 10^−3^
Biological Processes	Defense response to virus	8	80	2.42 × 10^−11^
	Defense response to symbiont	8	80	2.42 × 10^−11^
	Response to virus	8	80	2.91 × 10^−10^
	Type I interferon signaling pathway	6	60	2.00 × 10^−9^
	Cellular response to type I interferon	6	60	2.14 × 10^−9^
Pathways	Coronavirus disease—COVID-19	6	60	2.52 × 10^−8^
	NOD-like receptor signaling pathway	4	40	1.55 × 10^−4^
	Antiviral mechanism by IFN-stimulated genes	6	60	8.34 × 10^−10^
	Interferon signaling	7	70	1.08 × 10^−9^
	Cytokine signaling in immune system	8	80	7.65 × 10^−8^

**Table 5:** Top 10 compounds with high negative correlations for COVID-19–GBM association with respect to selected hub genes module and total DEG signatures.

Based on Hub Genes Module					
Top 10 Drugs					
S. No.		Score	Mechanism of Action	Status	Year of Approval
1	RUXOLITINIB	−0.89	JAK inhibitor	**Approved**	2011
2	LENALIDOMIDE	−0.86	Carcinogen	**Approved**	2005
3	PAZOPANIB	−0.83	VEGFR inhibitor|KIT inhibitor|PDGFR inhibitor	**Approved**	2009
4	PF-04457845	−0.83	FAAH inhibitor	Phase: 2	N/A
5	PRT-062070	−0.82	JAK inhibitor|Syk inhibitor	Phase: 2	N/A
6	TRAPIDIL	−0.81	PDGFR inhibitor	Phase: 1	N/A
7	EUGENOL	−0.81	Androgen receptor antagonist	**Phase: 1**	N/A
8	SR-59230A	−0.79	Adrenergic receptor antagonist	**Phase: 1**	N/A
9	UNC-669	−0.78	L3MBTL antagonist	**Phase: 1**	N/A
**Based on Total Differentially Expressed Genes**					
**Top 10 Drugs**					
1	Linezolid	−0.54	Bacterial 50S ribosomal subunit inhibitor	**Approved**	2000
2	Erastin	−0.54	Ion channel antagonist		N/A
3	Enalapril	−0.53	ACE inhibitor	**Approved**	1985
4	AZD-8055	−0.53	MTOR inhibitor	**Phase: 1**	N/A
5	Carbamazepine	−0.52	Carboxamide antiepileptic	**Approved**	1968
6	BX-795	−0.52	IKK inhibitor		N/A
7	Methotrexate	−0.52	Dihydrofolate reductase inhibitor	**Approved**	1953
8	Eugenol	−0.51	Androgen receptor antagonist	**Phase: 1**	N/A
9	Tolterodine	−0.51	Acetylcholine receptor antagonist	**Approved**	1998
10	Selumetinib	−0.51	MEK inhibitor	**Approved**	2020

## Data Availability

Not applicable.

## Supplementary Materials

The following supporting information can be downloaded at: https://www.mdpi.com/article/10.3390/ijms24043562/s1.

## Abbreviations

cMAP	connectivity map
overexpressed	up-regulated
low-expressed	down-regulated
DEGs	differentially expressed genes
GBM	glioblastoma
GEO	gene expression omnibus
GO	gene ontology
PPI	protein–protein interaction
KEGG	Kyoto encyclopedia of genes and genomes
Module	group of selected hub genes
